# Comparison of survival outcome of open, total laparoscopic, and laparoscopy-assisted radical vaginal hysterectomy for stage IB2 cervical cancer patients: A multicenter retrospective study

**DOI:** 10.1097/MD.0000000000037426

**Published:** 2024-03-08

**Authors:** Hyung Joon Yoon, Byung Su Kwon, Hyun Jin Rho, Tae Hwa Lee, Dae Hoon Jeong, Ki Hyung Kim, Dong Soo Suh, Yong Jung Song

**Affiliations:** a Department of Obstetrics and Gynecology, Pusan National University School of Medicine, Busan, Republic of Korea; b Biomedical Research Institute, Pusan National University Hospital, Busan, Republic of Korea; c Department of Obstetrics and Gynecology, Kyung Hee University Medical Center, Seoul, Republic of Korea; d College of Medicine, University of Ulsan, Ulsan University Hospital, Ulsan, Republic of Korea; e Department of Obstetrics and Gynecology, Kosin University Gospel Hospital, Kosin University College of Medicine, Busan, Republic of Korea; f Department of Obstetrics and Gynecology, Busan Paik Hospital, Inje University College of Medicine, Busan, Republic of Korea; g Department of Obstetrics and Gynecology, Pusan National University Yangsan Hospital, Gyeongsangnam-do, Republic of Korea.

**Keywords:** laparoscopy-assisted radical vaginal hysterectomy, overall survival, progression-free survival, stage IB2 cervical cancer, total abdominal radical hysterectomy, total laparoscopic radical hysterectomy

## Abstract

The aim of this study was to compare survival outcomes of 3 different radical hysterectomy (RH) types, namely total abdominal radical hysterectomy (TARH), total laparoscopic radical hysterectomy (TLRH), and laparoscopy-assisted radical vaginal hysterectomy (LARVH), in patients with FIGO stage IB2 cervical cancer. We retrospectively identified a cohort of patients who underwent RH for cervical cancer between 2010 and 2017. Patients with stage IB2 cervical cancer were included and were classified into TARH, TLRH, and LARVH treatment groups. Survival outcomes were estimated by the Kaplan–Meier method and compared with the log-rank test. Cox proportional hazards models were fit to estimate the independent association of RH technique with outcome. 194 patients were included in this study: 79 patients in the TARH group, 55 in the TLRH group, and 60 in the LARVH group. No significant differences were found in clinicopathological characteristics between the 3 RH groups. On comparing survival outcomes with TARH, both TLRH and LARVH showed no significant difference in terms of 5-year overall survival (TARH vs TLRH, *P* = .121 and TARH vs LARVH, *P* = .436). Conversely, compared to the TARH group, 5-year progression-free survival (PFS) was significantly worse in the TLRH group (*P* = .034) but not in the LARVH group (*P* = .288). Multivariate analysis showed that TLRH surgical approach (hazard ratio, 3.232; 95% confidence interval, 1.238–8.438; *P* = .017) was an independent prognostic factor for PFS in patients with IB2 cervical cancer. Our study suggests that in patients with FIGO stage IB2 cervical cancer, among the minimally invasive RH approaches, TLRH and LARVH, only TLRH approach was associated with worse PFS when compared with the TARH approach.

## 1. Introduction

Over the past 3 decades, there has been remarkable advancement in the field of minimally invasive surgery (MIS) for the treatment of early stage cervical cancer. Minimally invasive surgery radical hysterectomy (MIS RH), when compared to total abdominal radical hysterectomy (TARH), is related to reduced bleeding, shorter length of hospitalization, and decreased postoperative complications.^[1–5]^ A histological analysis revealed that MIS RH had no influence on the surgical extent on parametrial resection^[6]^ Furthermore, other research has demonstrated that MIS RH has survival outcomes that are equivalent to those of TARH.^[7–10]^ Previous findings indicate that MIS RH is commonly applied for the treatment of early stage cervical cancer. But, there is a limitation of level 1 data available for comparing the survival outcomes of the 2 RH procedures. Laparoscopic approach to cervical cancer (LACC) trial,^[11]^ a global phase III randomized trial, has reported that MIS RH was correlated with a significant decrease in disease-free survival (DFS) (hazard ratio [HR] 3.74, 95% confidence interval [CI] [1.63, 8.58]). Additionally, mortality was found to be 6 times higher (HR 6.00, 95% CI [1.77, 20.30]) when compared to TARH. Similarly, analysis of oncologic outcomes from the SEER database revealed that MIS RH has been associated with increased 4-year mortality rates.^[12]^ The findings of this analysis have caused widely debated on applying of MIS RH for early-stage cervical cancer.

The main problem is to uncover the probable risk related to the inferior oncologic outcome in MIS RH. Two recent retrospective studies comparing TARH and MIS RH reported similar disadvantages of oncologic outcome by MIS RH.^[12,13]^ However, sub-analysis of their study showed that MIS RH had worse outcomes than TARH only with cervical mass size > 2 cm in diameter but not with cervical mass size ≤ 2 cm. These findings suggest that large tumor size, which is already a well-known independent risk factor for early stage cervical cancer, may be a crucial risk factor for worse survival outcome, particularly with MIS RH.

Kohler et al assessed the oncologic outcomes of combined laparoscopic-vaginal radical hysterectomy (RH) in patients with initial FIGO stage identical to LACC.^[14]^ Several procedural steps in MIS, including the utilization of a uterine manipulator, intraperitoneal colpotomy, and carbon dioxide (CO_2_) pneumoperitoneum, have been suggested as potential risk factors related to a poorer survival outcome.^[15–17]^ Kohler et al reported the survival outcome of their surgical technique of combined laparoscopic-vaginal RH, in which they did not used uterine manipulator but created a tumor-covering vaginal cuff transvaginally. According to reports, the combined laparoscopic-vaginal RH has shown a 3-year overall survival (OS) rate of 98.5% and a DFS rate of 96.8%. These results are similar to the outstanding outcomes shown in the LACC for TARH. These data show the oncologic outcomes may differ according on the specific method of MIS RH.

All comparisons of the survival outcome between TARH and MIS RH have been investigated without distinguishing the type of MIS RH. With regard to the varying survival outcome with different MIS RH techniques, a comparative study of different types of MIS RH technique with TARH is required. In this study, we aimed to investigate the survival outcome of 3 surgical techniques—TARH, total laparoscopic radical hysterectomy (TLRH), and laparoscopy-assisted radical vaginal hysterectomy (LARVH)—in women with FIGO-2018 stage IB2 (2–4 cm) cervical cancer.

## 2. Materials and methods

### 2.1. Patients

The study cohort was obtained retrospectively from 3 hospitals in South Korea—Ulsan University Hospital and 2 Pusan National University Hospital (Pusan and Yangsan)—during the period from January 2010 to December 2017. The study protocol underwent revision and was subsequently approved by the institutional ethics committee of each respective institute. In this study, a total of 1328 women with primary cervical cancer who had surgical treatment with Piver type III or C1 RH were collected from a hospital record. Among of these, 194 women were collected based on the following criteria: FIGO-2018 stage IB2 disease^[18]^; no history of neoadjuvant chemotherapy; and no definite evidence of parametrial invasion and lymph node (LN) involvement in preoperative imaging findings. Additionally, cases of conversion from laparoscopic surgery to transabdominal surgery were excluded. Patients were grouped by 3 surgical techniques, namely TARH (n = 79), TLRH (n = 55), and LARVH (n = 60). The decision to perform MIS was made based on the surgeon’s preference considering the risk factors of the surgery and sharing decision making with the patient. When performing MIS for cervical cancer, TLRH was performed at Ulsan University Hospital and laparoscopic assisted radical vaginal hysterectomy was performed at Pusan National University Hospital.

The characteristics analyzed for each group included histology, tumor size, age, lympho-vascular space invasion (LVSI), LN metastasis, parametrial invasion, vaginal invasion, adjuvant treatment, time to disease progression, disease status and recurrence site. We classified cancer recurrence into 3 groups, A, B, and C: Recurrence A was defined as vaginal vault, intraperitoneal, and abdominal metastasis; Recurrence B was defined as pelvic, para-aortic, and other lymph node metastases; and Recurrence C was defined as liver, lung, and other hematogenous metastases.

### 2.2. LARVH techniques

Regardless of the extent of the tumor, a uterine manipulator (Humi® or Zumi™) was routinely used to enhance visibility of pelvic anatomy during laparoscopic surgery at our institution (Fig. 2). ZUMI uterine manipulator does not have a colpotomizer cup and is mainly used for laparoscopic uterine myomectomy or oopherectomy. As shown in Figure 2, we additionally mounted a balloon and used it in LARVH to reduce tumor bursting by minimizing the impact on the cervical mass. Laparoscopic resection of the parametria and pelvic lymphadenectomy were performed in all cases. The laparoscopy method included space development of the para-vesical area/para-rectal area, separating the ureter from the utero-vesical ligament, and surgically resecting the uterine vessels, as well as the middle portion of the utero-sacral ligaments. and The vaginal procedure was performed after irrigating the cervix with normal saline and evacuating CO_2_ gas intraperitoneally. The procedure consisted of cutting a 2 cm the vaginal cuff, and ligating the cardinal ligament.

**Figure 1.**
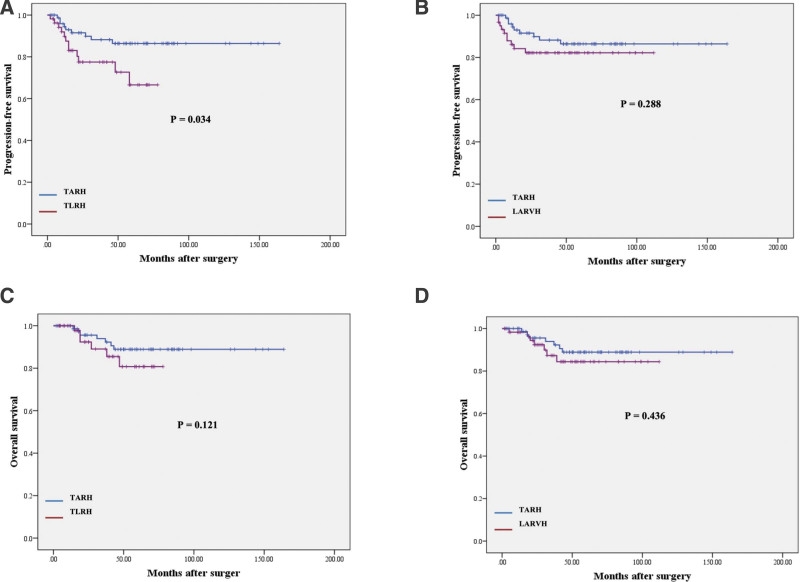
Comparisons of survival outcomes for patients with FIGO stage IB2 according to surgical approaches. TARH vs TLRH, (A) progression-free survival and (C) overall survival; TARH vs LARVH H, (B) progression-free survival, and (D) overall survival.

**Figure 2.**
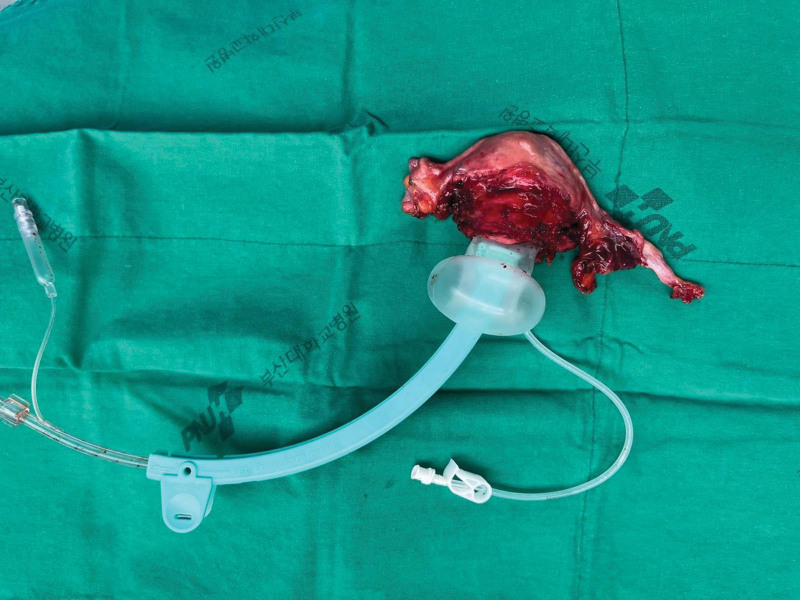
Uterine manipulator (Zumi™) to reduce tumor burst.

### 2.3. Statistical analysis

The Mann–Whitney *U* test and Student *t* test were used to compare continuous variables. The categorical variables were compared using Pearson chi-square test and Fisher exact test. The Kaplan–Meier method was utilized to calculate the 5-year progression-free survival (PFS) and OS. The differences in survival rates among the surgical technique groups were analyzed by the log-rank test. Multivariate analysis for disease recurrence was calculated using a COX regression model, and hazards ratio was calculated. A *P*-value < .05 was considered statistically significant. Statistical analyses were performed using SPSS Statistics 24.0 (IBM Corporation, Armonk, NY).

## 3. Results

### 3.1. Patients characteristics

Table 1 demonstrates the clinical features of the study population. The median age at diagnosis in the TARH group was 50 years, with a range of 26 to 74 years. The TLRH group had a median age of 52 years at the time of diagnosis, with a range of 35 to 75 years. The LARVH group had a median age of 51 years at the time of diagnosis, with a range of 26 to 85 years. There was no significant difference in the age among the various surgery groups (*P* = .774). The TARH group showed slightly higher rate of risk factors, including non-squamous cell carcinoma (SCC) histologic type, tumor size (≥3.0cm), LVSI, LN metastasis, parametrial involvement, and vaginal invasion than TLRH and LARVH (TARH vs TLRH vs LARVH: tumor mass ≥ 3.0 cm, 62.0% vs 58.2% vs 53.3%; non-SCC histologic type 22.8% vs 18.2% vs 20.0%; LVSI, 43.0% vs 30.9% vs 38.4%; LN metastasis, 25.3% vs 18.2% vs 20.0%; parametrial invasion, 13.9% vs 5.5% vs 8.3%; vaginal involvement, 5.0% vs 1.8% vs 1.7%). However, the differences in all risk factors among the 3 groups were not statistically significant (tumor size, *P* = .589; histologic type, *P* = .553; LVSI, *P* = .562; LN metastasis, *P* = .355; parametrial invasion, *P* = .241; vaginal involvement, *P* = .428). The TARH group showed a higher frequency of adjuvant therapy (TARH vs TLRH vs LARVH, 58.2% vs 41.8% vs 45.0%); however, there was no statistically significant difference among the 3 groups (*P* = .218).

**Table 1:** Patient characteristics based on surgical approach.

Characteristics	TARH (n = 79)	TLRH (n = 55)	LARVH (n = 60)	*P*
Age (years), median (range)	50 (2–74)	52 (35–75)	51 (26–85)	.774
*Tumor size (cm*), *mean* ± *SD*	3.7 ± 1.3	3.2 ± 1.1	3.0 ± 1.13	
<3.0	30 (38.0)	23 (41.8)	28 (46.7)	.589
≥3.0	49 (62.0)	32 (58.2)	32 (53.3)	
Histology type				
Squamous cell carcinoma	61 (77.2)	45 (81.8)	48 (80.0)	.553
Adenocarcinoma	17 (21.5)	9 (16.4)	9 (15.0)	
Other	1 (1.3)	1 (1.8)	3 (5.0)	
LVSI				
No	45 (57.0)	38 (69.1)	37 (61.6)	.562
Yes	34 (43.0)	17 (30.9)	23 (38.4)	
LN metastasis				
No	59 (74.7)	45 (81.8)	48 (80.0)	.355
Yes	20 (25.3)	10 (18.2)	12 (20.0)	
Parametrial invasion			0.109	
No	68 (86.1)	52 (94.5)	55 (91.7)	.241
Yes	11 (13.9)	3 (5.5)	5 (8.3)	
Vaginal involvement			0.703	
No	75 (95.0)	54 (98.2)	59 (98.3)	.428
Yes	4 (5.0)	1 (1.8)	1 (1.7)	
Adjuvant treatment			0.191	.218
No	33 (41.8)	32 (58.2)	33 (55.0)	
Yes	46 (58.2)	23 (41.8)	27 (45.0)	

Values are presented as median (range), mean ± standard deviation, or number of patients (%).

LARVH = laparoscopy-assisted radical vaginal hysterectomy, LN = lymph node, LVSI = lymph-vascular space invasion, TARH = total abdominal radical hysterectomy, TLRH = total laparoscopic radical hysterectomy.

### 3.2. Survival outcomes by RH techniques

The median follow-up duration in the TARH group was 82.1 months, ranging from 13.0 to 113.3 months. In the TLRH group, the median follow-up duration was 68.7 months, ranging from 17.5 to 102 months. Lastly, in the LARVH group, the median follow-up duration was 77.6 months, ranging from 22.1 to 115.6 months. The estimated 5-year PFS was 84.9% in the TARH group, 66.6% in the TLRH group, and 82.2% in the LARVH group. There were significant differences in PFS between TARH and TLRH groups (*P* = .034), whereas there were no significant differences between TARH and LARVH groups (*P* = .288) (Fig. 1A and B). In terms of OS, estimated 5-year OS was 88.9% in the TARH group, 88.9% in the TLRH group, and 88.1% in the LARVH group. There were no significant differences in OS (TARH vs TLRH, *P* = .121; TARH vs LARVH, *P* = .436 respectively) (Fig. 1C and D).

### 3.3. Recurrence patterns and risk factors

In the TARH group, 9 patients (11.4%) experienced a recurrence of cervical cancer, which affected 18 anatomical locations. In the TLRH group, 12 patients (21.8%) had a recurrence that affected 19 anatomical locations. In the LARVH group, 10 patients (16.7%) experienced a recurrence that affected 20 anatomical locations. Table 2 shows the recurrence patterns of cancer according to different surgical procedures. The rate of vaginal cuff recurrence was higher with both MIS TLRH (15.8%) and LARVH groups (20.0%) than with TARH groups (5.6%). The rate of intraperitoneal recurrence was the highest with TLRH groups (47.4%) among 3 types of surgeries (TARH, 27.8%; LARVH, 20.0%) (Table 2).

**Table 2:** Sites of recurrence based on surgical approach.

Location of recurrence	TARH (n = 9)	TLRH (n = 12)	LARVH (n = 10)
*A site*	6	12	8
Vaginal stump	1 (5.5)	3 (15.8)	4 (20.0)
Intraperitoneal cavity	5 (27.8)	9 (47.4)	4 (20.0)
*B site*	4	3	5
Pelvic or para-aortic LN	3 (16.7)	3 (15.8)	3 (15.0)
Other LN	1 (5.5)	0 (0.0)	2 (10.0)
*C site*	8	4	7
Lung or liver	6 (33.3)	3 (15.8)	5 (25.0)
Other	2 (11.1)	1 (5.3)	2 (10.0)
*Total no.*	18 (100)	19 (100)	20 (100)

Values are presented as number of recurrence (%).

LARVH = laparoscopy-assisted radical vaginal hysterectomy, LN = lymph node, LVSI = lymph-vascular space invasion, TARH = total abdominal radical hysterectomy, TLRH = total laparoscopic radical hysterectomy.

To estimate the independent association of RH techniques with PFS, multivariate analyses were performed in 2 different groups, TARH with TLRH and TARH with LARVH. In the TARH with TLRH group, LN metastasis (HR 3.252; 95% CI 1.207–8.756; *P* = .030) and TLRH (HR 3.232; 95% CI 1.238–8.438; *P* = .017) were independent poor prognostic factors for PFS (Table 3). Conversely, in the TARH with LARVH group, LN metastasis (HR 3.757; 95% CI 1.355–10.420; *P* = .011) was the only independent poor prognostic factor for PFS (Table 4).

**Table 3:** Univariate and multivariate analysis of patients with FIGO stage IB2 patients who underwent TARH (n = 79) and TLRH (n = 55) with regard to PFS.

Characteristics	Univariate analysis		Multivariate analysis	
	HR (95% CI)	*P*-value	HR (95% CI)	*P*-value
Age (years) (≥50 vs <50)	1.361 (0.577–3.206)	.481		
Tumor size (cm) (≥3.0 vs <3.0)	2.584 (0.946–7.056)	.064	1.914 (0.686–5.338)	.312
Histology (non-SCC vs SCC)	1.457 (0.565–3.756)	.436		
LVSI (yes vs no)	2.082 (0.884–4.904)	.093	1.378 (0.490–3.880)	.492
LN metastasis (yes vs no)	3.648 (1.549–8.593)	.003	3.252 (1.207–8.756)	.030
Parametrium invasion (yes vs no)	2.415 (0.809–7.207)	.114	2.200 (0.678–7.141)	.189
Vaginal margins positive (yes vs no)	1.420 (0.190–10.589)	.732		
Adjuvant treatment (yes vs no)	2.022 (0.816–5.015)	.128	1.180 (0.348–3.994)	.791
Surgical approach (TLRH vs TARH)	2.483 (1.040–5.930)	.041	3.232 (1.238–8.438)	.017

CI = confidence interval, FIGO = International Federation of Gynecology and Obstetrics, HR = hazard ratio, LN = lymph node, LVSI = lymph-vascular space invasion, PFS = progression-free survival, SCC = squamous cell carcinoma, TARH = total abdominal radical hysterectomy, TLRH = total laparoscopic radical hysterectomy.

**Table 4:** Univariate and multivariate analysis of patients with FIGO stage IB2 patients who underwent TARH (n = 79) and LARVH (n = 60) with regard to PFS.

Characteristics	Univariate analysis		Multivariate analysis	
	HR (95% CI)	*P*-value	HR (95% CI)	*P*-value
Age (years) (≥50 vs <50)	1.346 (0.547–3.314)	.518		
Tumor size (cm) (≥3.0 vs <3.0)	2.361 (0.850–6.557)	.099	2.035 (0.712–5.815)	.185
Histology (non-SCC vs SCC)	1.417 (0.510–3.936)	.504		
LVSI (yes vs no)	1.748 (0.703–4.348)	.229	1.283 (0.447–3.681)	.643
LN metastasis (yes vs no)	3.857 (1.567–9.496)	.003	3.757 (1.355–10.420)	.011
Parametrium invasion (yes vs no)	2.323 (0.770–7.009)	.135	1.771 (0.566–5.544)	.326
Vaginal margins positive (yes vs no)	1.316 (0.154–9.712)	.711		
Adjuvant treatment (yes vs no)	1.231 (0.485–3.128)	.662		
Surgical approach (LARVH vs TARH)	1.621 (0.658–3.996)	.294	1.659 (0.669–4.118)	.275

CI = confidence interval, FIGO = International Federation of Gynecology and Obstetrics, HR = hazard ratio, LAVRH = laparoscopy-assisted radical vaginal hysterectomy, LN = lymph node, LVSI = lymph-vascular space invasion, PFS = progression-free survival, SCC = squamous cell carcinoma, TARH = total abdominal radical hysterectomy.

## 4. Discussion

Although recent studies have raised doubts about the safety and effectiveness of MIS RH for early stage cervical cancer, it is important to analyze the probable factors that contribute to worse oncologic outcomes and develop viable solutions for MIS RH. J. Li. et al reported that when tumor size < 2 cm in 137 women with primary cervical cancer IA2-IB1, the oncologic outcome of TLRH was not inferior to that of TARH (the 5-year PFS: 96.4%, the 5-year OS: 96.8%). They tightened the vagina with cable ties to prevent tumor expulsion.^[19]^ As already mentioned, Kohler et al reports that the 3-year OS and DFS following combined laparoscopic-vaginal RH are 98.5% and 96.8%, respectively. In a previous study, we showed no significant difference in oncologic outcome between LARVH and TARH in cervical cancer 1A2-1B2 women.^[20]^ In this study, we assessed the oncologic outcome of 2 different MIS approaches for TLRH and LARVH by comparing them with TARH, in women with FIGO-2018 stage IB2 cervical cancer. Compared to the TARH group, the TLRH group demonstrated significantly worse PFS, consistent with LACC trial results, whereas LARVH did not. Furthermore, multivariate analysis showed that among MIS RH, only TLRH, along with LN metastasis, was identified as the risk factor for PFS in women with FIGO-2018 stage IB2 cervical cancer. The difference in PFS between TLRH and LARVH as observed in the present study indicates the presence of factors that influence the oncologic outcome during steps of MIS RH procedure.

Two different surgical procedures may be performed between TLRH and LARVH, each of which has the potential to affect oncologic outcome. One of those is the colpotomy; colpotomy was performed intraperitoneally in the TLRH group, but in the LARVH group, it was performed by vaginal approach. In addition, the TLRH group involved performing the intracorporeal colpotomy (IC) while the patient was in the CO_2_ pneumoperitoneum, whereas the LARVH group involved performing the vaginal colpotomy (VC) after removing the CO_2_. Kong et al performed a study to assess the recurrence rate in 128 women with FIGO-2018 stage IB and IIA cervical cancer who underwent laparoscopic/robotic radical hysterectomy (LRH/RRH). The study compared 2 different type of colpotomy: VC after CO_2_ removal (LRH-VC) and IC in CO_2_ pneumoperitoneum (LRH-IC).^[21]^ The retrospective study demonstrated that LRH/RRH-IC had a higher rate of disease recurrence in comparison to LRH/RRH-VC (16.3% vs 5.1%, *P* = .057). These findings suggest that performing IC in CO_2_ pneumoperitoneum may increase the probability of disease recurrence in MIS RH.

The LACC trial revealed variations in the recurrence patterns between TARH and MIS RH, with MIS RH being associated with a greater incidence of locoregional recurrence.^[11]^ In our series, vaginal cuff recurrence was more frequent in patients with both MIS, TLRH (15.8%) and LARVH (20.0%), than with TARH (5.5%) (Table 2). Furthermore, when comparing the rates of intraperitoneal recurrence, they were similar for TARH (27.8%) and LARVH (20.0%), but for TLRH (47.4%), they were relatively the highest among the 3 RH techniques (Table 2). This is also consistent with a previous Kong et al report which suggested that LRH/RRH-IC in CO_2_ pneumoperitoneum was related to higher rate of peritoneal recurrence (62%) than LRH/RRH-VC after CO_2_ removal (25%).^[21]^ These data support that performing IC in CO_2_ pneumoperitoneum is a significant predictive factor related to unfavorable survival outcomes in patients undergoing MIS RH.

Another difference is that although most MIS RH uses a uterine manipulator to improve visibility of pelvic anatomy, the type of uterine manipulator used is different in TLRH and LARVH. The TLRH procedure utilizes a manipulator that is attached with an extra colpotomizer, which helps to facilitate intraperitoneal colpotomy. In our study, the TLRH group used the RUMI® uterine manipulator and Koh® colpotomizer, and the LARVH group used Humi® or Zumi™ (Fig. 2). Using of a colpotomizer cup during the insertion of a uterine manipulator into the cervical OS complicates these procedures and leads to more manipulation of the tumor. This may lead to the perforation of a tumor in the cervix, which might cause the tumor to spread out, especially when vagina is resected and the tumor cell flow into circulating CO_2_. Kanao et al have emphasized the no-look no-touch strategy during TLRH.^[22]^ The no-look no-touch procedure in TLRH includes the formation of a vaginal cuff without requiring a uterine manipulator or substantial manipulation of the uterine cervix. Additionally, it includes the confinement of the specimen within a bag. There was no significant difference in the oncological outcome between TARH and TLRH when using this technique, even in cases in which the tumor size exceeded 2 cm. These findings show that surgical procedures for MIS RH should be performed without excessive handling of tumors.

Based on 2 recently published large studies from the USA, for MIS RH, tumor size is the most important prognostic factor. A tumor size larger than 2 cm was associated with poor prognosis for survival when operated upon laparoscopically.^[12,23]^ Similarly, a recent study in Korea reported that when compared with TARH, the recurrence rate after TLRH was equivalent to small tumors <2 cm but was significantly higher only in tumors larger than 2 cm.^[13]^ We also found that in patients with stage IB1 disease (tumor size < 2 cm), TLRH and LARVH were not associated with worse PFS (data not shown). These results suggest that MIS RH is feasible for selected patients with a tumor diameter < 2 cm.

Based on our results, we can speculate that this different risk of disease recurrence between 2 MIS RH could be due to IC in CO_2_ pneumoperitoneum and excessive tumor manipulation. Taken together, it can be inferred that trans-tumoral insertion of a uterine manipulator can cause the tumor to burst, and laparoscopic IC in CO_2_ pneumoperitoneum may result in exposure of these ruptured tumor cells to circulating CO_2_, thus leading to peritoneal tumor dissemination. Moreover, when the cervical tumor is large, it is predicted that these dangerous events are more likely to occur during MIS.

To our knowledge, this is first work to compare the survival outcome of 3 different RH approaches, TARH, TLRH, and LARVH, in early stage cervical cancer. Moreover, we included a homogeneous group with the stage IB2 cervical cancer (2–4 cm), which was previously reported as the high-risk group for survival outcomes in MIS RH, from a large series of patients. However there are some limitations associated with our study. First, it was a retrospective study. Second, the clinicopathological characteristics did not differ significantly between TARH and MIS RH groups, but TARH had a slightly greater susceptibility to risk factors, including larger tumor size (≥3.0 cm), non-SCC type, LN metastasis, LVSI, parametrial invasion, and vaginal invasion, leading to a higher frequency of requiring postoperative adjuvant therapy. This is because that this study was conducted retrospectively and the decision to perform TARH was based on the surgeon’s preference. Third, although it is a multi-center study, only one MIS RH procedure was performed in a single institute.

## 5. Conclusions

We find that in patients with IB2 cervical cancers, significantly worse PFS was observed in the TLRH group compared with the TARH group. TLRH was an independent prognostic factor for PFS in early stage cervical cancers. However, LARVH was not associated with worse survival outcomes. Comparison of surgical procedures between LARVH and TLRH led to the hypothesis that IC in CO_2_ pneumoperitoneum and excessive tumor manipulation increases intraperitoneal metastasis, which are preventable during MIS. Our findings, which have implications for the design of trial for MIS RH, need further examination in a new prospective randomized trial.

## Acknowledgments

This work was supported by a 2-Year Research Grant of Pusan National University.

## Author contributions

**Conceptualization:** Ki Hyung Kim, Yong Jung Song.

**Data curation:** Byung Su Kwon.

**Formal analysis:** Hyun Jin Rho.

**Investigation:** Dae Hoon Jeong.

**Methodology:** Tae Hwa Lee, Dong Soo Suh.

**Resources:** Tae Hwa Lee, Dae Hoon Jeong.

**Software:** Byung Su Kwon.

**Supervision:** Ki Hyung Kim, Yong Jung Song.

**Validation:** Hyung Joon Yoon.

**Visualization:** Hyun Jin Rho.

**Writing – original draft:** Hyung Joon Yoon.

**Writing – review & editing:** Dong Soo Suh.
